# Aging away from home: a cultural context and workforce challenges for African Australians; the necessity for an ‘Afrocentric’ care model in Australia

**DOI:** 10.3389/fpubh.2025.1577915

**Published:** 2025-04-30

**Authors:** Mimmie Claudine Watts

**Affiliations:** ^1^Institute of Health and Wellbeing, Federation University, Mount Helen, VIC, Australia; ^2^Office of the Pro Vice Chancellor FNU, Fiji National University, Fiji, Fiji; ^3^World Federation of Public Health Association, Geneva, Switzerland; ^4^African Science Research and Innovation Council (ASRIC), African Union Commission, Addis Ababa, Ethiopia

**Keywords:** migrant workforce, social capital, cultural capital, filial care, intersectionality, employment, older persons, Black Australians

## Abstract

This perspective examines the emerging African Australian older adult group, a minority group within a minority and the need for an Afrocentric-oriented workforce. It discusses aging and support systems within a traditional African setting, contrasted to the solitary state of the aging African Australian diaspora, while highlighting the presence of a CALD workforce ready to support their older adults. Intergenerational coresidency, a cultural practice common in African family structures, like Asian communities, are shared. The values of respect, communal existence, filial responsibilities, and the need for connections to kin by older adults are explored. The challenges facing older adult parents left behind in Africa, while their children search for better opportunities abroad, need addressing. Protracted issues of limited participation in the workforce earlier in life, and its subsequent impact on the individual’s later life, have profound financial impact on them. In conclusion, gaps in the workforce to meet the needs of an older adult minority group are sought. A proposed Afrocentric workforce framework to provide assistance to older adults is examined and proposed.

## Introduction and overview

As the aging demographics expand in multicultural Australia (1), and globally, the situation of aging African-born migrants living in separation from extended family is becoming increasingly relevant (2, 3). In Australia, people of African ancestry number over 300,000 (4), though when the second and third generations are included, that number becomes closer to half a million. The evolving demographic landscape of older adults globally necessitates urgent attention to address the workforce challenges in elder care among Australians from a predominantly minority background (1, 4–6). In 2016, for example, there were approximately 1,000 Sub-Saharan born African Australians aged 65 and over. This figure is projected to expand to 29,000 persons. That is a + 2,245% growth by to 2056. The only other comparison group with such exponential growth is Central Asia estimated to grow by +3,783% from 3,000 to 59,000 (36). This highlights the need for services and a workforce to care for this emerging group of older Australians using cultural inclusive care models, as they move into residential aged care; a transition away from the traditional intergenerational coresidency in Africa. Among these challenges, language barriers stand out as a significant impediment (7, 8). Effective communication is crucial for the delivery of quality care, yet linguistic discrepancies can lead to misinterpretations of medical needs and lack of understanding of the emotional well-being of older adults (9).

The necessity to cultivate a workforce that reflects the cultural values and experiences of the African diaspora cannot be overstated (10–12). Having a workforce with shared linguistic and cultural similarities will enhance the care experiences of this population group, despite individual differences. Training healthcare professionals who are acquainted with African cultural norms surrounding aging can lead to enhanced experiences for both caregivers and care recipients. This community-centric approach to elder care integrates respect, dignity, and holistic well-being, which are pivotal in addressing the needs of older adults. To ensure services that meet the needs of older adults from this community, an understanding of aging within a traditional African structure is necessary (13).

With an aging population, the emerging older adult African Australian community requires a workforce that understands their cultural nuances, and their intersectional existence as a minority group in Multicultural Australia. Many people from this minority migrant group experience lower SES, with direct impact to later life experiences. Known economic disparities, and low socioeconomic status that exists among migrants impacts negatively on their wellbeing and therefore requires attention and action (14). A culturally competent workforce with in-depth knowledge of African cultures and values toward older adults is essential to meet the needs of older adults (2, 12).

This informed opinion narrative outlines the workforce needs, to help close the disparity wellbeing gap for aging African Australians (15, 16). It is constructed from first hand interactions of the author with African Australian communities of which she had been a member for a quarter of a century. It draws on her knowledge of African cultures and customs, on her interactions, on her travels to multiple African countries and settings, and on their migration histories, and includes her own knowledge as an academic. The author, however, acknowledges her own ‘privileged’ position as a skilled professional migrant and a university professor.

This account attempts to provide context on the aging experience in a ‘natural’ African setting: the support systems, the informal workforce, and the networks. It highlights challenges, while providing strategies to help improve the wellbeing of the aging African Australians (6, 12). It seeks backing to help support a needed Afrocentric workforce. It solicits for political and structural support for African Australian migrants to foster connections with kin in the homeland of birth, particularly as they age.

The narrative highlights the benefits of filial support and the need for a ‘workforce’ that understands the collective African value systems (3, 17). It seeks to highlight policy gaps, with the aim of gaining the support of policy makers to implement policies that address the issues of isolation and poorer health status for African Australians (1, 18, 19). This will ensure that aging African Australians receive culturally appropriate care, centered in the values of Afrocentrism, regardless of setting (10).

### Aging in an African traditional context, a familial workforce

Older adult individuals living alone, or in an aged care facility (nursing home), remain a novel situation in Africa and to most Africans. It was not a part of the African culture until recently. It’s becoming a social issue of concern for the older adult person and for African communities, in Africa as well as abroad (2, 12, 16, 20). Unlike their older adult African counterparts who often enjoy a sense of belonging provided by immediacy to family, many Australian seniors are confined to solitary living arrangements (18). In Australia, the reverence that was often given to older individuals in African cultures is frequently diminished, leading to poorer health outcomes (12, 18). The wisdom and life experiences that they possess are often not recognized, valued, nor validated. This cultural shift can result in further isolation and emotional detachment for older adult migrants, resulting in a decrease in their sense of being and their traditional identity (19).

The cultural shift from multigenerational living to solitary aging in Australia is detrimental to the health of older African Australians (7, 9). This isolation is compounded by systemic issues in the aged care sector (1). Further, the increasing reliance on paid home care services to provide assistance to older adults often lacks the personal warmth and cultural sensitivity present in familial care (15). Although older migrants may initially seek assistance from paid professionals, the presence of cultural and linguistic barriers can lead to feelings of dissatisfaction (19). Paid care often comes without the familial connection that signifies love and respect in their home communities. This loneliness causes mental health issues such as depression and anxiety for both the workers and older adults (15). The absence of traditional support mechanisms means that individuals often lack the practical assistance to assist with chores at home, historically available from family members in African countries (12, 20). These care responsibilities have often relied heavily on female members of the family (8).

### Intergenerational living and coresidency

African cultures, like Asian societies, encourage intergenerational coresidency (3). This practice is underpinned by filial support, with the aim to support aging parents and older adults (8, 14, 21, 22). Intergenerational living or coresidency with family encourages and supports extended family members to live together. Culturally, older adults age at home among familiar extended family members, including their sons and daughters, sons-in-law and daughters-in-law, grandchildren, and often great grandchildren. Extended family members ensure that elders, depending on health status and agility, participate in light household chores of their choosing (13). These keep them physically and cognitively engaged.

The presence of multiple generations within the same setup is an asset. Nurturing, watching over their grand and great grandchildren is considered a noble job. Children, for example, may obtain water from the tap, at home, or from a nearby stream, and grandparents in turn shower the children with love, praises, and a great deal of wisdom. These enhance the family bonds. The parents of the grandchildren, being themselves the children to these grandparents, find joy supporting both generations (21). This ‘workforce’ doubles as a social support system for older adults. Caring for one’s older adult parents is not considered ‘work,’ nor a ‘sacrifice,’ and it’s believed to bring with it blessings.

Such a ‘natural’ structure not only enhances emotional bonding but also facilitates caregiving through a shared responsibility model (8); a model in which everyone knows their role. Coresidency provides a mutually beneficial workforce for grandparents, their children and grandchildren (8); a structure that everyone belongs to and contributes to, for the benefit of the family unit.

For example, grandparents are peace makers and play key roles during social ceremonies such as commemoration of births, marriages, and anniversaries (21). These occasions serve as fundamental cultural spheres binding families and communities together (3). The ‘children’ (in the middle) who are also ‘parents’ to their own children, provide support to both groups. While this middle group is commonly referred to as the ‘sandwich generation’ in Australia, and to an extent in OECD countries, this ‘sandwich’ arrangement of caring for older adults and one’s own children, has and remains part of the ‘Ubuntu’ African family structure. It’s not a strain; rather, it’s an honor that only some have, seeing their parents attain old age.

Positive experiences of such multigenerational living arrangements are well documented (13, 20, 22). However, coresidency by migrant families in developed countries has received mixed views (2, 14), compounded by the poorer mental health and low financial status for those who relocated to new homelands later in life (2, 3, 14). Co-residency is partially or completely absent post relocation from Africa to Australia, breaking the cycle of filial piety. Further, African cultures emphasize the role of the family and community in the care of elders, wherein caring for aging parents is viewed as a sacred responsibility (13, 21).

Filial Piety on the other hand imparts a sense of duty for children to care for their aging parents (12). This attitude reflects a commitment to honor, respect, and support the elders, an African cultural practice identical to Asian and South American cultures (6, 8, 20, 21, 23). Respect for parents and older adults remains a fundamental principle in African cultures. This is because African cultures value and emphasize respect for elders, as they acknowledge the wisdom gathered through life experiences. They value communal sharing of responsibilities, while emphasizing ties with kin even when individuality is present (23). Such an approach contributes to a cultural holistic care environment for older adults, with benefits to its workforce.

### Holistic familial support, respect, versus aging in isolation

Holistic care, being an essential aspect of wellbeing, focuses on the mental and social wellbeing of the older adult person and has proved to be beneficial for intergenerational coresidency. It reflects the strengths of the family and of grandparents, particularly grandmothers, who often possess the unique multidimensional positions of storyteller, minder, family therapist, spiritual mentor and leader (8, 21). As such, respect for older adults remains a cornerstone of African cultures, regardless of setting or blood ties to the individual (8, 20, 21). Respect toward older adults is expected but not demanded.

Aging and becoming an ‘elder’ in African society was, and is, considered arriving at a new ‘status’ in one’s life, viewed in positive light (23). This period was accompanied by devoted communal support, intergenerational residency, and co-dependency, with financial ‘freedom’ another benefit for those with successful children. Unfortunately, expatriation is eroding these customary systems (21, 24).

With the emigration of children to western countries in search of better opportunities, the increasing shift in traditional African coresidency and co-dependency structures is deteriorating, in Africa and abroad. This is much to the disadvantage of the older adult parent(s) (13). This also erodes the central position of grandparents within their families and, to an extent, in the community. With their children living and working abroad, parents are aging alone in Africa, regardless of the financial benefits from their children abroad (13). Conversely, aging in a ‘new land’ and away from home, as a minority older adult person, brings with it complex challenges, including loneliness (2, 8, 12).

Many older African migrants are confronted by profound loneliness, exacerbated by cultural shifts in care expectations from children, and the erosion of traditional family systems, further burdened with financial responsibility and systemic neglect in their new homelands (12, 13, 18, 20, 22). Victimhood becomes a reality for some of these elders, although they refuse to view themselves as such; a narrative common among the Australian Indigenous Elders (25). Like Australian Indigenous elders, a minority group within a minority, older adult African Australians are becoming a minority within a minority group.

Challenges ranging from lack of ‘respect,’ language barriers, limited knowledge of services and rights are common (1, 6, 12, 17). The implications of aging in isolation are profound. Not only does it affect the physical and mental health of older African migrants, but it also has broader societal ramifications. The erosion of kinship ties and community engagement threatens to undermine the social fabric that traditionally sustains and nurtures older family members, detracting from the dynamic intergenerational exchanges that can enrich the entire community.

### Dislocation of the ‘filial’ workforce

The migration of African youth to western countries, is destabilizing sociocultural family dynamics, stripping seniors, and relatives of a natural lively ‘workforce’ (2). The youth are also impacted as they will also become older adults at some point. Aging African migrants in Australia experience a disconnect, with geographical and cultural barriers resulting in significant isolation, exacerbated by the distance (12). For example, the visitor visa restrictions imposed by the Australian Immigration and Home Affairs Departments on the migrant person’s kin, impact negatively on aging African Australian migrants and their relations. Visiting relatives in Australia is quite a tedious and expensive process compared to Europe and North America, where frequent multiple entry visitor visas to parents are often supported.

### Disenfranchisement

Economic disruption and migration stressors contribute to the challenges migrants face, especially for those accustomed to communal systems of support (19). Local knowledge and social networks are also contributors to successful integration and resettlement (26). The critical lack of traditional support networks and the fractured kinship ties, exacerbated by limited resources at a perilous stage in life, raises concerns for this group (19). This phenomenon is not isolated to African Australians; it has been observed among other aging African diaspora from Senegal, Eritrea, and Egypt in their ‘new homelands’ of Italy (2).

Within the Australian diaspora, the ‘new emerging’ aging African population is burdened with economic obstacles, underpinned by integration challenges, further compounded by low English language proficiency, underemployment, and structural racism (17). This group consist primarily of folks who arrived as refugees in the late 1980s and early 1990s from the Horn of Africa (24). Barriers to social inclusion, employment and career advancement exist, due to ‘covert’ entrenched racism against migrants; more so, against Black persons (5).

Institutional structural racism, often based on accent, skin color, hair texture etc., has been reported as a barrier to employment, adequate housing, and career advancement within Australian multicultural communities (27). These barriers have hindered Australians, particularly visibly ‘coloured’ migrants, from full participation in the workforce, with significant economic impact. This results in high unemployment rates, close to 50% in some African Australian communities, compared to the 4% Australian national average (28). This then has resulted in high welfare dependency and at-risk behaviors for young people, often under-or unemployed.

The limited recognition of qualifications gained overseas reduces employment opportunities, often resulting in lower skill jobs, lower income, and financial difficulties later in life for migrants. It is well established that the skills sets and roles held during one’s working life contribute to their income and superannuation. Superannuation is a long-term investment that increases over the course of time and builds toward your retirement. The retirement amount you receive is co-relational to what you contribute during your working life; the more you will have for retirement. In Australia, superannuation is compulsory (29). Full and meaningful involvement in the workforce, therefore, have a direct correlation to superannuation available at retirement for the individual. Additionally, superannuation is expected to last for the entire duration of one’s retirement. It is unfortunate that this is not a positive experience for African Australians due to limited workforce participation earlier in life.

Unfortunately, the superannuation balance of migrants is lower than that of their Australian born counterparts of the same age (30), though there are minor exceptions for skilled migrants who spoke English as their primary language pre-migration. Females within these migrant groups are worse off comparatively, due the existing gender pay gap that advantages men. This often leaves older migrant women with inadequate resources to experience a meaningful retirement. Thus, employment and full participation in the workforce immediately following relocation is essential for later life. A workforce that has insights and direct experiences of such migration nuances is essential. Support toward aging African Australian diaspora necessitates support from a knowledgeable workforce familiar with African cultures.

## Policy implications

### The need for an African Australian diaspora elder care workforce and Afrocentric models

The presence of migrants across the care and services sectors is well documented (15). The number of Culturally and Linguistic Diverse (CALD) background individuals employed in the aged care sector in 2020 was 49,475, accounting for 35% of the residential age care workforce. What was even more fascinating was that they were more likely to work in residential facilities with more CALD clients (31). African Australian diaspora, a minority CALD group, form part of this Residential Age Care (RAC) workforce. Their continued commitment to care for their elders at all levels of service is crucial. Regardless, the overrepresentation of CALD and African Australian workforce in the aged care sector and other care settings, needs to be recognized, supported and enhanced. This will ensure that care providers acquire valuable cultural norms and customs from their older adult kin, while older adults receive cultural holistic care; a mutually beneficial reciprocal approach for both.

To enhance the presence of African diaspora in the elder care workforce, several strategies may be employed. For example, have role models of African heritage who are successful leaders in the sectors. This will encourage more engagement and participation from the community. Highlighting the impact of their work and its benefit to their aging community can strengthen ties within these groups. This can be enhanced through the provision of targeted scholarships to African Australian diaspora youth, to complete programs that lead to employment within the aged care sector, with clear career pathways. This will reduce unemployment, while ensuring a good superannuation when the individual eventually retires.

An Afrocentric approach, which matches people of African backgrounds with kin will benefit aging African Australians. A pilot community led initiative, pairing volunteers of similar backgrounds with older adults, is proving to be successful (32). Afrocentric values can be embedded in service provision using modern technologies (33). Further, as a communal group, African community leaders’ involvement in decision-making that impacts the lives of the older adult African Australians will enhance service delivery. This can be achieved through civic involvement, while leveraging the social and youth capital from existing ‘natural’ groups and from volunteers, preferably from the same ethnic group. It can foster resilience among carers/volunteers, thereby improving the experience for service recipients and carers (34).

Communication and meaningful connections remain common obstacles to full participation in the workforce (9, 16). Initiatives that foster intergenerational connections and that facilitate engagement can alleviate loneliness for the older adult person and carer, while enhancing the service delivery experience (2, 33). Initiatives should include relevant programs and activities for older adults, by carers from similar cultural and linguistic backgrounds. To alleviate the language barriers in various caregiving settings, Afrocentric cultural competency training (35), audiovisual multilingual resources, enhanced ethnic technological solutions, and regular engagement with families and community should be adapted and utilized. The use of modern technologies will enhance the experience (33).

Language and literacy programs tailored to older migrants can empower individuals to navigate the Australian care systems more effectively (6). Furthermore, collaboration between governmental agencies, local communities, and organizations serving African migrants are essential to cultivate knowledge and develop inclusive frameworks for the aged care sector that respect and honor their cultural identities, underpinned by human rights (27). Fostering open channels of communication with family and other professionals in Australia and abroad will enhance feelings of connection with kin, thus bringing positive emotions for both carers and older adults. Using existing techniques to enhance cultural sensitivity among the health and other workforce, could be utilized to the residential age care sector, particularly those with clients from CALD.

### Limitation

This is an informed ethnographic narrative and opinion piece, which is informed by the authors’ experiences, observations, extensive readings, travels, a lengthy academic career of two decades, and as a member of the African Australian community. This should be considered when interpreting this narrative.

## Conclusion

Members of the African Australian diaspora are an essential component of multicultural Australia, with their presence visible across almost every sector, despite the challenges. With an aging African Australian diaspora, it is necessary to understand essential African cultural norms, such as co-residency, intergenerational living, filial support, and respect for older adults. These have traditionally boosted older adults’ wellbeing as they age. The challenges of aging African migrants in Australia reflect not only their personal struggles, but also a broader societal issue that requires immediate attention. Policymakers must understand the value of kinship networks and community-based support systems.

A multi-dimensional approach is required to address workforce issues in elder care, particularly in communities where the African diaspora are prevalent. By incorporating African cultural values into training, and by engaging the diaspora in caregiving roles, a more inclusive, respectful, and effective elder care system can be developed to provide cultural holistic care. This approach does not only honor the inherent dignity of older adults but also revitalizes the communal aspect of caring for elders, a principle that holds significant importance in African cultures. An Afrocentric oriented workforce can assist in that. By recognizing their unique experiences and fostering comprehensive support systems, Australia can better accommodate the needs of older African migrants, ensuring they are accompanied by dignity, respect, and a sense of belonging.

## Data Availability

The original contributions presented in the study are included in the article/supplementary material, further inquiries can be directed to the corresponding author.
